# The effect of the use of a decision aid with individual risk estimation on the mode of delivery after a caesarean section: A prospective cohort study

**DOI:** 10.1371/journal.pone.0222499

**Published:** 2019-09-26

**Authors:** Emy Vankan, Ellen Schoorel, Sander van Kuijk, Jan Nijhuis, Rosella Hermens, Hubertina Scheepers, on behalf of the SIMPLE study group

**Affiliations:** 1 GROW-School for Oncology and Developmental Biology, Department of Obstetrics and Gynaecology, Maastricht University Medical Centre+, Maastricht, The Netherlands; 2 Department of Clinical Epidemiology and Medical Technology Assessment (KEMTA), Maastricht University Medical Centre+, Maastricht, The Netherlands; 3 Scientific Institute for Quality of Healthcare (IQ healthcare), Radboud Institute for Health Sciences (RIHS), Radboud University Medical Center Nijmegen, Nijmegen, The Netherlands; Foundation IRCCS Neurological Institute C. Besta, ITALY

## Abstract

**Objective:**

After one previous caesarean section (CS), pregnant women can deliver by elective repeat CS or have a trial of labor which can end in a vaginal birth after caesarean (VBAC) or an unplanned CS. Despite guidelines describing women’s rights to make an informed choice, trial of labor and VBAC rates vary greatly worldwide. Many women are inadequately informed due to caregivers’ fear of an increase in CS rates in a high VBAC rate setting. We compared counseling with a decision aid (DA) including a prediction model on VBAC to care as usual. We hypothesize that counselling with the DA does not decrease VBAC rates. In addition, we aimed to study the effects on unplanned CS rate, patient involvement in decision-making and elective repeat CS rates.

**Methods:**

We performed a prospective cohort study. From 2012 to 2014, 483 women in six hospitals, where the DA was used (intervention group), were compared with 441 women in six matched hospitals (control group). Women with one previous CS, pregnant of a singleton in cephalic presentation, delivering after 37 weeks 0 days were eligible for inclusion.

**Results:**

There was no significant difference in VBAC rates between the intervention (45%) and control group (46%) (adjusted odds ratio 0,92 (95% Confidence interval 0.69–1.23)). In the intervention group more women (42%) chose an elective repeat CS compared to the control group (31%) (adjusted odds ratio 1.6 (95% Confidence interval 1.18–2.17)). Of women choosing trial of labor, in the intervention group 77% delivered vaginally compared to 67% in the control group, resulting in an unplanned CS adjusted odds ratio of 0,57 (0.40–0.82) in the intervention group. In the intervention group, more women reported to be involved in decision-making (98% vs. 68%, P< 0.001).

**Conclusions:**

Implementing a decision aid with a prediction model for risk selection suggests unchanged VBAC rates, but 40% reduction in unplanned CS rates, increase in elective repeat CS and improved patient involvement in decision-making.

## Introduction

After one previous caesarean section (CS), pregnant women can deliver by elective repeat caesarean section (ERCS) or have a trial of labor (TOL) which can end in a vaginal birth after caesarean (VBAC) or an unplanned CS. Despite guidelines describing women’s rights to make an informed choice and that for most women vaginal birth is safe [1–3], TOL and VBAC rates vary greatly worldwide [4]. In the United States (US), the VBAC rate is around 8.3% [5]. In 2015, 1.3 million women in the US underwent a CS (32%) [6], making it one of the most frequently performed operative interventions in women. In The Netherlands, VBAC rates are much higher (54%) [7], but most women are inadequately informed about their choices due to caregivers’ fear of an increase in CS rates [8]. Morbidity risks (both maternal and neonatal) are directly related to the individual probability of VBAC since morbidity is lowest in women with VBAC, but higher in women with unplanned than planned CS [9]. We developed a decision aid (DA) containing evidence based information including a prediction model [10, 11]. A better inventory of risks, benefits and chance of success might improve shared decision making and reduce unplanned CSs without bringing down the number of VBAC.

We aimed to evaluate the effect of counseling with a decision aid (DA) including a prediction model compared to regular counseling without the DA on the VBAC-rate. We hypothesize that counseling with the DA does not decrease VBAC rates (i.e. non-inferiority with respect to VBAC rate). In addition, we aimed to study the effects on unplanned CS rate, patient involvement in decision-making, ERCS rate, guideline adherence and maternal and neonatal complications.

## Materials and methods

### Study design & setting

We performed a prospective cohort non-inferiority study: we hypothesised that counseling with the DA does not decrease VBAC rates. In a preliminary study in 2010 [12], VBAC and TOL rates in 17 hospitals in the Netherlands were studied as part of the SIMPLE I study. For this current study (SIMPLE II), twelve of these hospitals were selected based on type of hospital (university, teaching and non-teaching hospitals) and their 2010 VBAC rates (<percentile (p) 20, p20-80 and >p80) and divided into six matched pairs based on these criteria. The groups had a comparable VBAC rate in the pre-measurement (46 vs 53%, non-significant). In the period between September 2012 and September 2014, we applied our DA in six of the hospitals (intervention hospitals) in one region, and compared VBAC rates and the secondary outcomes with the six matched hospitals providing counseling without the DA (control hospitals) (S1 File).

This design was chosen based on different facts. There is close collaboration between hospitals and midwifery practices in a small country as the Netherlands. Midwifery practices may work with several hospitals in one region. In this study setting referring midwives would not have to deal with two policies, with chance of bias. Besides, caregivers like residents who work in different settings in one region during their specialization were not biased by knowledge of the use of the DA when they switch to another hospital.

The study was a consecutive study of the previously reported SIMPLE study [8]. The study was initially designed as a controlled before and after study with an intervention trial incorporated in the second part. When reporting the results, this created an overcomplicated method section with no added value to the results. In this final design, data from the before measurement were used to select two comparative groups of hospitals (S1 Table). In this setting the prospective intervention trial was performed, improving clarity of the study.

### Study population

All women with a singleton pregnancy and a foetus in cephalic position, delivering after 37 weeks after one previous CS, without a contra-indication for a TOL, were eligible for inclusion. The DA was developed as a tool to improve guideline adherence. Therefore the Medical Ethical Committee of the MUMC+ agreed that no individual informed consent was needed for receiving the DA (the DA was assigned at hospital level). The DA was administered to all eligible women in only the intervention hospitals. Informed consent was only needed and obtained from the patients (with command of the Dutch language), who were willing to complete the questionnaire.

### Intervention

We developed a DA for mode of delivery after previous CS including a prediction model for predicting the individual probability of a VBAC. The content was based on literature search, expert’s opinion and the contemporary international guidelines on delivery after previous CS [11]. Seven steps essential for decision-making in mode of delivery were included: preferences for mode of delivery before reading the DA (1), previous birth experiences (2), risks and benefits of TOL and ERCS (including the prediction model)(3), a worksheet to weigh out the options (4), a birth plan where women can write down their preferences (5), preliminary choice (6) and follow-up to reevaluate the decision (7). The DA was developed according to the IPDAS criteria and pilot tested in 25 women.

A woman’s individual probability on a VBAC was calculated with a previously developed prediction model [10]. This model was developed for a West-European population and includes indication for previous CS, body mass index (BMI), need for labor induction, ethnicity, estimated fetal weight in the current pregnancy and a previous vaginal delivery. Our database was too small to determine a cut-off level in individual probability on VBAC and in temporary literature there is no evidence for a specific cut-off level. For this reason we did not present a cut-off level, giving women a possibility to weigh their own probability. Women in the intervention hospitals received the DA before 36 weeks of pregnancy were able to read and discuss it with relevant others before discussing the mode of delivery with their obstetrical caregiver. During consultation around 36 weeks the DA was used as guidance of the counseling for mode of delivery and shared decision making and the prediction model was filled in. The woman’s individual probability on a VBAC was then showed in a percentage. At this point a shared decision was made on the mode of delivery. During this consultation women were asked for informed consent for completing the questionnaire send by email. The counseling was repeated if the circumstances changed (for example when labor induction was needed). In each intervention hospital, an one-hour training for healthcare professionals was organized to discuss the content of the DA and to instruct how to use the DA in daily practice.

In the control hospitals, patients received according to the contemporary guideline oral and/or written information on mode of delivery after CS as usual from their obstetrician or midwife during regular consultations in their pregnancy. According to the contemporary Dutch guideline women should be informed about the chance of a VBAC, the risk of a uterine rupture, the increased risk of a uterine rupture in use of oxytocin or prostaglandins, the risks and advantages of an ERCS, the implications for a possible next pregnancy and the policy in case of spontaneous labor. The discussion on mode of delivery also took place around 36 weeks during consultation. The DA and the prediction model were not used in the control group.

### Outcome measures

The primary outcome was the VBAC-rate. Secondary outcomes were unplanned CS rate, patient involvement in decision-making, ERCS rate, guideline adherence for counseling and maternal and neonatal complications. Patient involvement was defined as a positive answer to the question if a woman felt involved in the decision making regarding the choice of mode of delivery. This question was part of a validated questionnaire, used for other studies as well [13].

Guideline adherence was defined as mentioning in the patient chart discussing the following items: risk of uterine rupture, the increased risk of uterine rupture when using oxytocin or prostaglandins, the chance of VBAC, and risk of perinatal mortality. Maternal complications included more than 4 packed cells, uterine rupture, hysterectomy, operational injury, thrombosis, maternal death and admission to the intensive care unit. Neonatal complications included neonatal death, neonatal asphyxia, plexus lesion of fracture and admission to the neonatal intensive care.

### Sample size

We hypothesised an equal VBAC rate between the intervention and control group and considered a decrease in VBAC rate of over 10% in the intervention group undesirable. Therefore, a non-inferiority limit of 10% was chosen. Consequently, a VBAC rate of more than 10% reduction in the intervention group compared to the control group would be seen as ‘inferior ‘care. In 2010 the SIMPLE study showed a national VBAC rate of 49% [12]. The necessary sample size for showing non-inferiority, with an alpha of 0.05, a beta of 0.20, and an intra-cluster correlation coefficient (ICC) of 0.2, was 400 per study-arm.

Each hospital was asked to prospectively include 80 consecutive eligible women. The participating hospitals were requested to administer questionnaires to a convenience sample of 30 of the 80 eligible patients during the study-period. This sample size was based on a needed number for measuring decisional conflict also measured with this validated questionnaire [13] also used for other studies. The Ottawa Health Decision Centre indicates that an effect size of 0.3–0.4 is meaningful [14]. We chose an alpha of 0.05, a beta of 0.20 and a standard deviation of 17.5 [15]. Therefore, for detecting an effect size of at least 0.3, a difference between DC scores of 5.25 between the intervention group and the control group was needed. We estimated the ICC to be 0.2. The estimated sample size, corrected for cluster variation, was 200 per study arm.

### Data collection

Data on VBAC, unplanned CS and ERCS were collected from the medical records and national perinatal registry by trained staff using case report forms. A validated questionnaire was used to ask among other things, whether patients felt involved in the decision making regarding the choice of mode of delivery [13]. The questionnaire was handed out to the patients online at 37 weeks of pregnancy after informed consent. Patients were instructed to fill in the questionnaire after discussing and having decided upon mode of delivery with their obstetric caregiver. The variables of guideline adherence (risk of uterine rupture, the increased risk of uterine rupture when using oxytocin or prostaglandins, the chance of VBAC, and risk of perinatal mortality) were measured according to their frequency of documentation in the medical records. Data on maternal complications(more than 4 packed cells, uterine rupture, hysterectomy, operational injury, thrombosis, maternal death and admission to the intensive care unit) and neonatal complications (neonatal death, neonatal asphyxia, plexus lesion of fracture and admission to the neonatal intensive care) were extracted from medical records and the national perinatal registration. Information on actual use of the DA, demographic factors, obstetric history, and current pregnancy (co-variables) to check if the intervention group and control group were comparable, was extracted from medical records and the national perinatal registry.

### Statistical analyses

Data analysis was carried out using SPSS 21. All consecutive eligible women were included in the statistical analysis, irrespective of their actual exposure to the DA. Only the women, who gave informed consent for the questionnaire, received the questionnaire. The data were entered in the study database by study staff. In case of inconsistencies, data were checked with the hospital concerned. Missing covariable data were imputed using stochastic regression imputation, since complete case analysis may decrease statistical power and induce bias if data are not ‘missing’ completely at random.

In order to study the effects of the DA, data analysis was performed according to intention-to-treat and per-protocol principle (women actually receiving the DA). Non-inferiority was analysed by converting the non-inferiority limit of 10% (in relative risk) to a limit in odds ratio (OR) on VBAC in the intervention group compared to the control group. The OR’s for VBAC were calculated by multivariable regression analysis, correcting for the variables of the prediction model and age. To check for non-inferiority, the 95% confidence interval (CI) of the OR was then assessed with the non-inferiority limit. If the 95% CI limits were exceeding the non-inferiority limit, non-inferiority cannot be proven. The same procedure for calculating OR was followed for the unplanned CS rate. Differences in patient involvement and guideline adherence between women in the intervention and control group were tested using chi-square. The relative risks estimates for complications were calculated using cross-tabs.

### Details of ethical approval

Ethical approval for this study was obtained from the Medical Ethical Committee of Maastricht University Medical Centre+ in The Netherlands (MEC number 12-4-091) (25-07-2012). The study started in September 2012. Local approval was received from the boards of directors of local hospitals. Written informed consent was obtained from the patients willing to complete the questionnaire. The DA was developed as a tool to improve guideline adherence. Therefore the Medical Ethical Committee of the MUMC+ agreed that no individual informed consent was considered needed for this part of the study.

### Trial registration

This study is registered at ClinicalTrials.gov: *Current Dutch Practice on Caesarean Sections*: *Identification of Barriers and Facilitators for Optimal Care (SIMPLE)*, NCT01261676, https://clinicaltrials.gov//show/NCT01261676?term=cesarean&rank=18

## Results

### Baseline characteristics

Table 1 shows that baseline characteristics of the women in the intervention- and control-group were mostly comparable. In the intervention group women were a bit younger and had a shorter pregnancy, less women had a previous CS due to failure to progress and more women had a previous vaginal birth, however this difference was not significant.

**Table 1:** Baseline characteristics.

	Intervention group (N = 483)			Control group (N = 441)			p-value/test
	N/Mean	%/range/SD	Missing	N/Mean	(%/range/SD	Missing	
**Demographic** **factors**							
Caucasian, (%)	395	82,2%	3	343	78,3%	3	0,14/chis
Maternal age (years)	33,33	**±**4,35	6	33,9	**±**4,53	24	0,06/t-test
BMI (kg/m^2^)			67			94	*
<20	44	10,6%		32	9,2%		0,55/ chis
20–24,9	165	39,7%		142	40,9%		0,77/ chis
25–34,9	174	41%		149	42,9%		0,90/chis
>35	33	7,9%		24	6,9%		0,68/ chis
**Obstetric history**							
Parity	1,32	0,77	2	1,29	0,68	3	0,83/ MW
Indication previous CS failure to progress	192	44,1%	48	201	47,1%	14	0,41/ chis
Previous vaginal birth	91	19,0%	5	73	16,7%	3	0,39/ chis
**Current pregnancy**							
Gestational age (in days)	276,20	8,09		277,23	7,83		0,044/MW
Estimated fetal weight from GA 32 weeks p≥90	27	6,4%	59	33	7,6%	6	0,51/ chis
Induction of labor	90	21,5%	65	94	25,4%	71	0,21/ chis*

SD: standard deviation, chis: chi-square, BMI: body mass index, MW: Mann-Whitney-U test, CS: caesarean section, GA: gestational age, p: percentile

* missing data > 10%

### Main outcome

**Fig 1:**
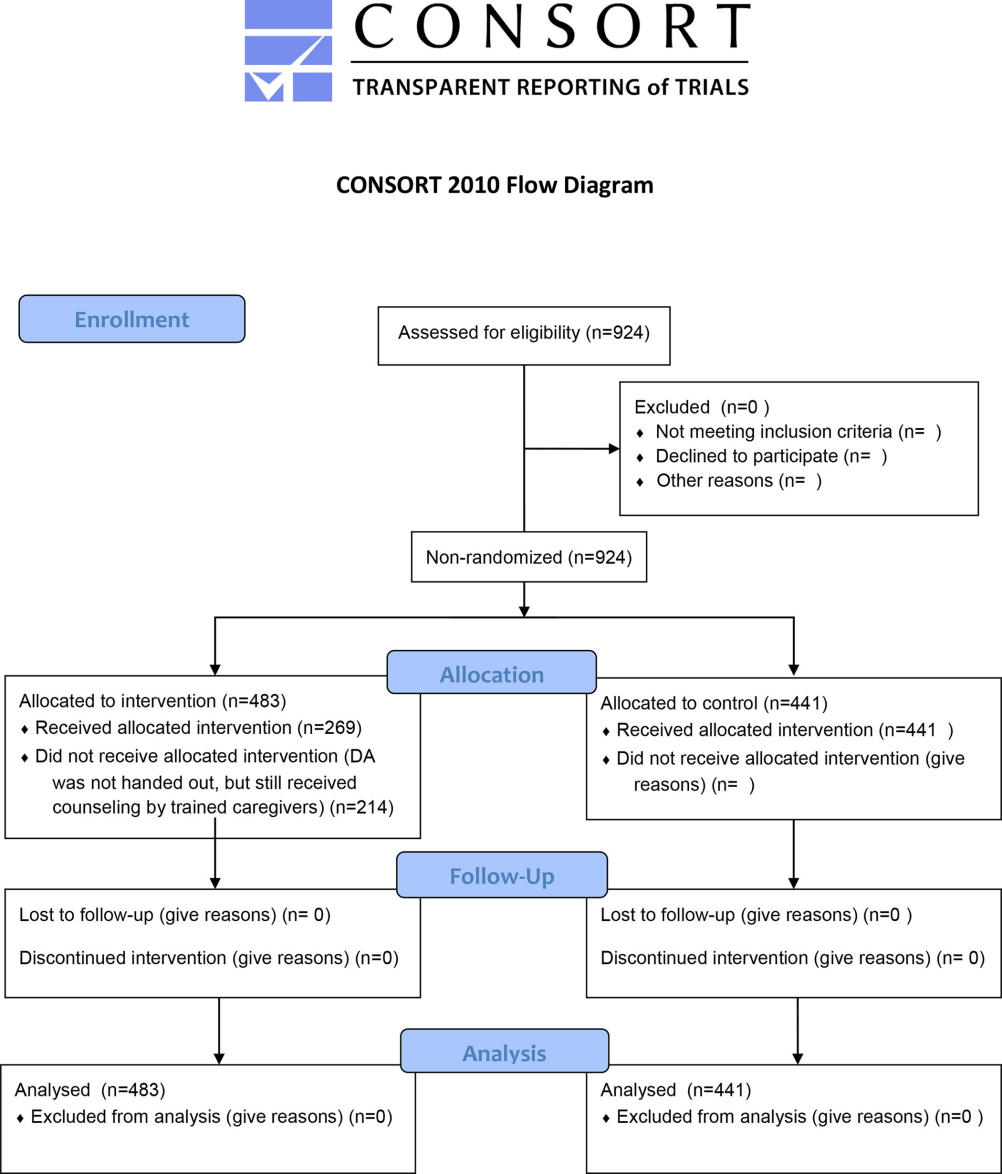
Consort flow diagram. Enrollment, allocation, follow-up and analysis.

We analyzed 924 women, 483 in the intervention group and 441 in the control group, respectively (Fig 1). All consecutive eligible women were included in each hospital, except for one control hospital due to bankruptcy (45 women included). No women were excluded. There was no difference in VBAC rates between the intervention and control group (45 vs. 46%). The crude odds-ratio (OR) for VBAC for the intervention group compared to the control group, was 0,96 (95% CI 0,74–1,24). When correcting for differences in predictors of the prediction model in both groups, the adjusted OR was 0,92 (95% CI 0.69–1,23) (Table 2). We also corrected for other possible predictors of VBAC: pre-eclampsia or the syndrome of hemolysis, elevated liver enzymes, and low platelet count (HELLP), hypertension and diabetes in the current pregnancy and maternal age based on previous studies [10], with unchanged outcome.

**Table 2:** Mode of delivery outcome.

	Intention to treat						Per-protocol DA vs. control group			
	Intervention (N = 483) (%)		Control (N = 441) (%)		Crude OR (95% CI)	Adjusted OR (95% CI) (corrected for risk factors)	Intervention (N = 269) (%)		Crude OR (95% CI)	Adjusted OR (95% CI) (corrected for risk factors)
**Mode of delivery**										
Elective repeat caesarean delivery	201	41,6%	137	31,1%	1,6 (1,21–2,07)	1,6 (1,18–2,17)	112	41,6%	1,6 (1,16–2,17)	1,6 (1,14–2,32)
Spontaneous delivery	182	37,7%	167	37,9%	0,99 (0,76–1,30)	0,95 (0,71–1,28)	108	40,1%	1,1 (0,81–1,50)	1,1 (0,76–1,51)
Instrumental delivery	35	7,2	36	8,2%	0,88 (0,54–1,42	0,91 (0,57–1,48)	22	8,2%	1,0 (0,58–1,74)	1,0 (0,58–1,79)
Unplanned caesarean section	65	13,5%	101	22,9%	0,52 (0,37–0,74)	0,57 (0,40–0,82)	27	10,0%	0,38 (0,24–0,59)	0,41 (0,26–0,66)
Intended vaginal birth (trial of labor)	282	58,4% (282/ 483)	304	68,9% (304/ 441)	0,63 (0,48–0,83)	0,63 (0,46–0,85)	157	58,4%	0,63 (0,46–0,87)	0,62 (0,43–0,88)
Vaginal birth of those with a trial of labor	217	76,9% (217/ 282)	203	66,8% (203/ 304)	1,7 (1,15–2,39)	1,4 (0,96–2,12)	130	82,8%	2,4 (1,49–3,87)	2,0 (1,17–3,28)
**Total VBAC rate**	217	44,9% (217/ 483)	203	46,0% (203/ 441)	0,96 (0,74–1,24)	0,92 (0,69–1,23)	130	48,3%	1,1 (0,81–1,49)	1,1 (0,77–1,50)

OR: odds ratio, CI: confidence interval, VBAC: vaginal birth after caesarean

To test non-inferiority for VBAC, we used a non-inferiority limit of 10% (RR 0.9 in case of a decrease in VBAC) which was converted into a limit in OR. Based on a prevalence of 46% of VBAC in the control group, the non-inferiority limit for the OR was 0.83. The 95% CI of both the crude OR (0.96 (95% CI 0.74–1.24)) and the adjusted OR (0,92 (95% CI 0.69–1,23)) for VBAC crossed the non-inferiority limit in the intention to treat analysis. Therefore, even though the point estimate suggests that the use of the DA did not decrease VBAC beyond the non-inferiority limit, the results are not conclusive because of the wide confidence intervals. This occurred in the per-protocol analysis as well, where the crude OR was 1.1 (95% CI 0.81–1.49). In the intervention-group 269/483 (56%) of the women received the DA. In the per-protocol analysis the VBAC rates were comparable as well (48% vs. 46%). The predicted chance of succeeding in VBAC was comparable in women who received the DA and who did not receive the DA in the intervention group (p = 0,95).

### Secondary outcomes

Table 3 shows the results of the different onsets of delivery. In the intervention group more women chose an ERCS (42%) compared to the control group (31%), (adjusted OR 1.6 [95% CI 1.18–2.17]) (intention to treat analysis). Of the women starting a TOL, in the intervention group the vaginal delivery rate was higher (77%), compared to the control group (67%) (adjusted OR 1.4 [95% CI 0.96–2.12]) (Table 2). Subsequently, even after correction, the unplanned CS rate was lower in the intervention group (14%) compared to the control group (23%) (adjusted OR 0.57 [95% CI 0.40–0.82]). The per-protocol analysis showed similar results; the adjusted OR for an unplanned CS in the intervention group was 0.41 [95% CI 0.26–0.66]. In total 130/269 women chose for a TOL and 83% had a VBAC compared to 67% in the control group (adjusted OR 2.0 [95% CI 1.17–3.28]).

**Table 3:** Onset of delivery outcome.

	Intention to treat						Per-protocol DA vs. control group			
	Intervention (N = 483) (%)		Control (N = 441) (%)		Crude OR (95% CI)	Adjusted OR (95% CI) (corrected for risk factors)	Intervention (N = 269) (%)		Crude OR (95% CI)	Adjusted OR (95% CI) (corrected for risk factors)
Onset labor										
Elective repeat caesarean section	201	41,6%	137	31,1%	1,6 (1,21–2,07)	1,6 (1,18–2,17)	112	41,6%	1,6 (1,16–2,17)	1,6 (1,14–2,32)
Spontaneous onset	184	38,1%	207	46,9%	0,70 (0,54–0,90)	0,58 (0,43–0,78)	100	37,2%	0,67 (0,43–0,88)	0,60 (0,42–0,86)
Priming with balloon catheter	45	9,3%	35	7,9%	1,2 (0,75–1,89)	1,8 (1,01–3,05)	21	7,8%	0,98 (0,56–1,73)	1,4 (0,68–2,67)
Priming with prostaglandins	3	0,6%	1	0,2%	2,8 (0,29–26,54)	3,1 (0,29–32,20)	2	0,7%	3,3 (0,30–36,52)	3,6 (0,31–41,37)
Induction	50	10,4%	61	13,8%	0,72 (0,48–1,07)	0,77 (0,46–1,29)	34	12,6%	0,90 (0,58–1,41)	0,82 (0,44–1,50)

OR: odds ratio, CI: confidence interval, VBAC: vaginal birth after caesarean

Regarding decision making, 256 out of 367 women (n = 205 in intervention group and n = 162 in control group), filled in the questionnaire. Unfortunately, not all caregivers handed out the questionnaire and not all women replied, in spite of several reminders. In the intervention group 133 out of 137 (98%) stated they were involved in the choice for the mode of delivery, compared to 78 out of 119 (68%) in the control group (p< 0.001).

Table 4 shows the guideline adherence results based on intention to treat analysis. In the intervention group, counseling on mode of delivery was more frequently reported in the medical record (73% vs. 63%, p< 0.001), as were the specific required items (p<0.05). Of course, in a per-protocol analysis, all women received adequate information.

**Table 4:** Guideline adherence.

	Intention to treat			Per protocol		
Items guideline adherence	Intervention group (N = 483)	Control group (N = 441)	p-value	Intervention group (N = 269)	Control group (N = 655)	p-value
**Counseling documented in medical file**	353(73%)	278 (63%)	p = 0.01	269 (100%)	362 (55,3%	p<0,001 p<0,001
**Chance of VBAC discussed**	295 (61,1%)	141(32,0%)	p<0,001	269 (100%)	167 (25,5)	p<0,001
**Small risk of uterine rupture discussed**	288 (59,8%)	183 (41,5%	p<0,001	269 (100%)	202 (30,8%)	p<0,001
**Risk perinatal death discussed**	282 (58,4%)	118 (26,8%)	p<0,001	269 (100%)	131 (20%)	p<0,001
**Increased risk of uterine rupture in use of oxytocin or prostaglandins discussed**	286 (59,2%)	185 (42,0%)	p<0,001	269 (100%)	202 (30,8%)	p<0,001

VBAC: vaginal birth after caesarean

Table 5 shows the complication rates, which overall were low. When analyzing the total major complication rate, no significant differences were seen in uterine ruptures, perinatal deaths and asphyxia. The incidence of neonatal infection and the necessity for antibiotic treatment in women postpartum was comparable as well.

**Table 5:** Incidence major complications.

	Intervention group (n = 483) (%)	missing	Control group (n = 441) (%)	missing	Relative risk
**Major maternal complications**							
> 4 packed cells	0	0%	7	1	0,2%	0	Na.
Uterine rupture	6	1,2%	0	2	0,5%	0	2,7 (0,56–13,50)
Hysterectomy	0	0%	0	0	0%	0	Na.
Operational injury	0	0%	0	2	0,5%	1	Na.
Thrombosis	0	0%	0	1	0,2%	2	Na.
Maternal death	0	0%	0	0	0%	0	Na.
Admission ICU	1	0,2%	7	2	0,5%	3	0,46 (0,04–5,06)
**total**	7			8			
Patients with 1 or more maternal complications	7	1,5%	10	6	1,4%	6	1,1 (0,36–3,17)
**Major neonatal complications**							
Neonatal death	0	0%	0	2	0,5%	2	Na.
Neonatal asphyxia	20	4,1%	5	20	4,6%	4	0,91 (0,50–1,66)
Plexus lesion or fracture	0	0%	5	0	0%	2	Na.
Admission NICU	9	1,9%	15	11	2,5%	7	0,76 (0,32–1,81)
**total**	29			33			
Patients with 1 or more neonatal complications	26	5,6%	16	25	5,8%	11	0,96 (0,56–1,63)
**Total major complications**	36			41			
**Patients with 1 or more major complications**	31	6,7%	21	27	6,4%	17	1,05 (0,64–1,74)

Na.: not applicable, ICU: intensive care unit, NICU: neonatal intensive care unit

## Discussion

### Main findings

This study showed that using the DA for the counseling on mode of delivery, does not result in a change in VBAC rates in a setting with a high VBAC rate. Less women chose a TOL, however the undesirable event of an unplanned CS was much less common. Although statistically non inferiority could not be proven and a change in VBAC incidence cannot completely be ruled out, we found an important shift from unplanned to planned CS. Women receiving the DA and choosing TOL in the intervention group succeeded more often than women choosing TOL in the control group. Women who received the DA felt more involved and were more often counseled according to the guidelines. This shows that the implementation of our DA results in a better risk-based choice of delivery and a more personalized counseling.

### Strengths and limitations

This is the first study evaluating the use of a DA with a prediction model for mode of delivery after CS in a prospective, comparative setting. Several prediction models concerning vaginal birth after CS have been developed in the past [16–29]. However, none has been tested in practice as part of counseling. The DA itself was earlier tested according to the International Patient Aid Standards (IPDAS) and met 39/50 criteria [11, 30] because effectiveness measurements were not performed yet. By using it in a prospective setting the effectiveness can be evaluated as well. Taking this into account, the DA meets 46/50 IPDAS criteria [30]. Furthermore, our study included a large group of patients from different types of hospitals.

The design of our study was a prospective cohort study. Selection of the intervention hospitals was based on geographical region and VBAC results in usual care, which may cause bias. The reason this design was chosen instead of a cluster randomized trial is the fact that in a small country as the Netherlands, there is close collaboration between hospitals and midwifery practices. In this study setting referring midwives would not have to deal with two policies. Besides, caregivers like residents who work in different settings in one region during their specialization were not biased by knowledge of the use of the DA. A randomized controlled trial on patient level was not possible due to effects on knowledge and information bias by caregivers.

Only 56% of the women in the intervention group received the DA, which can be explained by the fact that caregivers did not have sufficient attention to the DA in spite of instructions. To study the effect of the intervention as a whole and specifically the use of a DA, we performed both per-protocol analysis of the women who actually received the DA, and an intention-to-treat analysis showing that the effects were highest in women actually receiving the DA, but also were present in women that did not. This means that also effects on hospital level are present suggesting improved knowledge of caregivers or the use of the prediction model without the DA. As a result of the lack of compliance in the intervention group, differences between the groups are likely attenuated, leading to an lower statistical power than anticipated. However, Table 2 shows that most clinically relevant associations, quantified as odds ratios, are accompanied by relatively small confidence intervals, and differences with respect to guideline adherence (Table 4) are all highly significant. This shows that the study still had sufficient power to detect meaningful differences between groups.

Only 256 out of 367 women who received the questionnaire, returned it, in spite of several reminders.

Although underpowered, no difference in the total incidence of complications between the two groups was observed. In general, complication risks are expected to be higher in an unplanned CS compared to elective CS a [9]. A benefit of using the DA on complication rate has to be studied in future research.

### Interpretation

International guidelines stress that individual characteristics and preferences of the patient should be taken into consideration when counseling for mode of delivery [1–3]. The individual probability for a VBAC is a main determinant for the risk of severe maternal morbidity and an important factor in the decision making process. This study showed that women in the intervention group choosing for TOL more often succeeded in VBAC. This might be an effect of the calculated individual probability, leading to a choice for TOL when the chance on VBAC is high. This would contribute to better risk selection. The effect of the DA and the prediction model was analyzed as one single intervention. Consequently, it is difficult to determine whether the results are due to the prediction model or improved counseling. However, according to the temporary guidelines women should be informed about their chance on VBAC. Discussing their individual probability is therefore inseparable from adequate counseling. There was no selection bias in women who received the DA, when the predicted chances in both groups were similar. One of our future studies will focus on the impact of the use of the prediction model.

A DA is an effective instrument to support the shared decision-making process and helps patients to make an informed choice. In our study women who received the DA felt more involved in the decision making, however this was not tested in the before-setting. Stacey et al. showed that patients felt more involved in the decision making process when a DA was used [31]. Shorten et al. showed increased knowledge and less decisional conflict in patients using a DA [32]. Montgomery et al. also showed reduced decisional conflict in women after using a DA, but no clear differences in mode of delivery [33]. Horey et al. reported no difference in preferred mode of delivery after interventions for supporting decision making [34]. Gardner et al. showed an increase in VBAC after implementation of two other strategies: a standing team of three high-risk consultants and a antenatal clinic for counseling women for mode of delivery after caesarean during three visits [35]. To the best of our knowledge this is the first study showing a change in not only the preferred mode of delivery but also in the actual mode of delivery after the use of a DA (more ERCS, less unplanned CS). These differences can be explained by the risk selection due to the incorporation of the prediction model. Besides the role in the decision making process, the DA can help caregivers to inform patients according to the guidelines. In spite of the implementation of our intervention and a rise in counseling according to the guideline, there is still no complete guideline adherence in the intervention group. This might be due to the fact that the use of the DA was still in the implementation phase and the fear of caregivers for a rise in CS rate when counseling adequately.

The effects of the intervention in countries like the US, with an a priori low VBAC-rate are unclear. Patients possibly have other preferences, but particularly the cultural background and the preference of the healthcare providers might play a role. Besides, in this study, most of the women were Caucasian, who are more likely to delivery vaginally compared to Hispanic or African American women [4]. However, it is not unlikely that in countries with a lower VBAC rate or other ethnicities, the use of a DA may also lead to a distinct decrease in CS.

### Conclusions

In a setting with a high VBAC rate, a patient DA for mode of delivery after CS, appear to result in similar VBAC rates, but further research is necessary to prove non-inferiority. However, we found a higher successful TOL a lower unplanned CS rate and a higher ERCS rate. This suggests an improved risk selection.

## Supporting information

S1 File Study protocol. Cesarean Section IMPLEmentation (SIMPLE) II Study.
(DOC)

S2 File TREND checklist (Transparent Reporting of Evaluations with Nonrandomized Designs).
(PDF)

S3 File Database. (SAV)

S1 Table Overview of VBAC-rates per hospital before and after the intervention per hospital. (DOCX)
