# Supplementary material for: The effect of the use of a decision aid with individual risk estimation on the mode of delivery after a caesarean section: A prospective cohort study
Source: PLoS One. 2019 Sep 26;14(9):e0222499. doi: 10.1371/journal.pone.0222499 (PMC6763212; doi:10.1371/journal.pone.0222499)
Supplement: S1 File — Cesarean Section IMPLEmentation (SIMPLE) II Study. (DOC) [file pone.0222499.s001.doc]

**Cesarean Section IMPLEmentation (SIMPLE) II Study**

| **Projectleaders**  Dr. H.C.J. Scheepers, Maastricht UMC+  Dr. R.P.M.G. Hermens, IQ Healthcare Nijmegen | **PhD students**  Drs. S. Melman, Maastricht UMC+  E.N.C. Schoorel, MD., MSc., Maastricht UMC+  Drs. E. Vankan Maastricht UMC+ | **Projectmembers**  Prof. Dr. J. G. Nijhuis, Maastricht UMC+  Dr. L. Smits, epidemiologist, Maastricht University  Prof dr. C.D. Dirksen, Maastricht University  Prof. Dr. T van der Weijden, Maastricht University  Dr. A. Kwee, UMC Utrecht  Drs. S.M. van Kuijk, Maastricht University |
| --- | --- | --- |

**Contact:**

Drs. E Vankan

Maastricht Universitair Medisch Centrum+

P. Debyelaan 28

6229 HX Maastricht


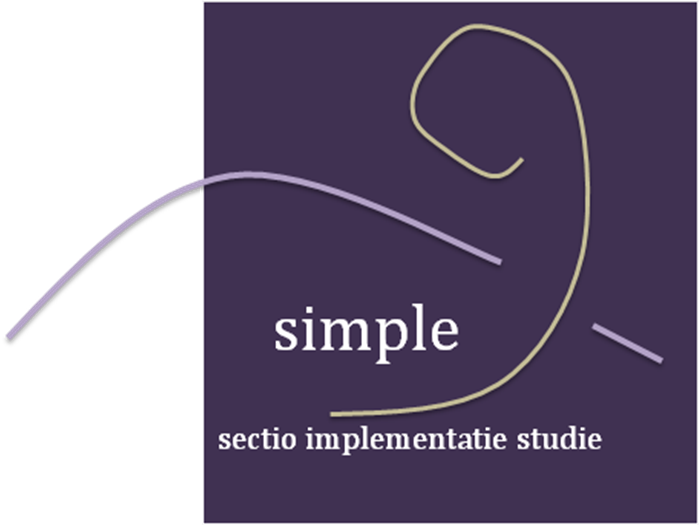
Tel: 043 3874768

E-Mail: emy.vankan@maastrichtuniversity.nl

General information SIMPLE study: [www.studies-obsgyn.nl/simple](http://www.studies-obsgyn.nl/simple)

**A controlled-before and after trial for increasing guideline adherence regarding counseling mode of delivery after previous cesarean section**

1. **Background**

Pregnant women with a previous cesarean section (CS) have to decide on mode of delivery in their current pregnancy. The options are a repeat CS, an intended vaginal birth after cesarean (VBAC). An intended VBAC results in 70-75% in a vaginal delivery and consequently results in 25-30% in an (emergency) CS. When no contra-indication for an intended VBAC exists, both options should be offered. The aim of the current study is to evaluate whether our decision aid (DA) is effective for increasing adherence to guideline-based quality indicators while not leading to a decrease in VBAC-rate.

**Rationale**

The Section IMPLEmentation study (SIMPLE) study measured current Dutch care on CSs by comparing current care with optimal care as described in national and international guidelines. The indicators with the lowest guideline adherence were indicators on counseling of pregnant women with a previous CS. In this group, the guideline adherence was only 4-15%. The frequency of occurrence was 2.2-11.7%. In general 11.7% of the pregnant women visiting the obstetrics department had a history of previous CS.. Also, there was an inter-hospital variance-rate for intended VBAC of 55-95%. This large practice variation could not be explained by variability in patient characteristics and indicated that currently there is no uniformity in policy between hospitals. Interviews with most of the patients perceived current provision of information as insufficient. In interviews with providers it appeared that providers experience uncertainty when counseling women on mode of delivery, because they fear that provision of more information will lead towards an increase of the repeat CS rate.

**Patient decision aid**

Mode of delivery after previous CS should ideally be discussed in a shared decision making setting. For shared decision making, a patient decision aid (DA) could be a good instrument. DA’s have proven to be effective tools for supporting the decision making process [1]. Two studies that intervene with a DA on the matter of mode of delivery after CS, have shown to reduce decisional conflict and increase knowledge [2,3]. Both studies did not lead to an increase of repeat CSs or total CS rate [2,3], in fact the study of Montgomory et al [3], showed an increase of total VBAC-rate. These findings are consistent with the results of the Cochrane review by Stacey et al., who reported a reduction of elective surgery in favor of conservative treatment options when a patient DA was used for counseling [1]. The effect on altered information supply in a setting with an a priori high intended VBAC rate is however, unclear. It is even imaginable that increased information supply in this setting increases the repeat CS rate. Therefore, evaluation on both CS rates as patient and caregivers experiences is necessary.

Based on literature, the results of analysis of current Dutch care and patient opinions, we chose to develop a patient DA for counseling on mode of delivery after one previous CS for the Dutch setting. Compared to the above mentioned studies, this DA was particularly developed to enhance adherence to guideline-based quality indicators with regard to counseling on mode of delivery after previous CS. The DA was developed according to a systematic development process. The content of the DA was based on an extensive literature search, expert’s opinions and the Dutch guideline in practice on CS after previous CS: Dutch society of obstetrics and gynecology (NVOG): ‘pregnancy and delivery after a previous cesarean section 04-06-2012’. The framework was selected according to the checklist of the International Patients Decision Aid Standards (IPDAS) criteria. The DA was pretested in 25 women of our target group, in order to optimize clearness and usability.

Further, we added a prediction model for predicting the probability of a successful intended VBAC to the patient DA. In comparison to the previous published DA’s for mode of delivery after previous CS, this prediction model is a new item. General VBAC rates worldwide are 60-80% [5], in the Netherlands this currently is 72% [6]. However, these rates are less applicable for the individual woman since women with a higher or lower probability of success may be counseled different. The probability of an intended VBAC resulting in a VBAC is considered important for counseling because it is a main determinant for the risk of severe maternal morbidity. The lowest incidence of major maternal morbidity is observed in women with VBAC (0.2%), followed by women with a repeat CS (0.8%) and unsuccessful intended VBAC (3.8%) [7]. We assumed that the outcome of the prediction model would be important for the decision making process. During patient interviews in the pre-testing phase of the DA systematic development process, this assumption was confirmed since all women stated that they considered their probability of a VBAC relevant for their decision on mode of delivery. We developed the prediction model for a West-European population of women with one previous CS. This model was based on the prediction models by Grobman et al. [8,9]. The strengths of the VBAC prediction models of Grobman et al. are that they were developed in a large cohort of 7,660 and 9,616 women and had already been successfully validated in an independent cohort [10,11]. These models were developed for an American population women with a previous CS, for the Western European population, the two models were combined based on applicability of predictors. Also, to increase validity, the models were recalibrated and variables were redefined. Further, the variable estimated fetal weight (EFW) was added to the model in order to increase its applicability for obstetrical care. The final prediction model consists of six variables: 1) Estimated Fetal Weight > p90; 2) Recurrent indication of CS 3) previous vaginal delivery; 4) Induction; 5) Body Mass Index and 6) European. The model was internally validated with a bootstrapping procedure. The discriminative capacity of the model was obtained by measuring the area under the curve (AUC) of the Receiver Operating Characteristic (ROC) curve. The AUC of the prediction model was 70.8% (95% confidence interval = 68.6 – 72.9%), indicating reasonable discriminative capacity. The calibration plot showed a good predictive capacity in all ranges. The model was considered suitable for implementation into clinical practice because of two main reasons: 1) the model was based on two prediction models that already have shown to be valid for an external population and 2) internal validation of the model showed good results.

We will evaluate the process of implementing the DA for patients and providers. Patients receive a questionnaire with questions concerning satisfaction, clarity and applicability of the DA and their experience with Shared-Decision-Making. The experiences of providers are collected in focus groups. During these focus groups , training, satisfaction, applicability, clarity, recruitment and limiting factors are discussed.[12] Finally we will analyze the costs for developing and implementing the DA and evaluate possible costs of shift in CS rate.

In conclusion, we expect that implementing a patient DA in current Dutch obstetrical care, will not lead to the undesirable event of a raise in repeat CSs but to a more patient-centered care, in which women can make an informed decision on mode of delivery after previous CS. Due to the possibility of prediction of the probability of VBAC, we hypothesize that the patient DA could in fact induce a shift in preference of women for desired mode of delivery after previous CS. This effect could lead to a reduction of the emergency CS rate while the VBAC rate is maintained, indicating a reduction of major maternal and neonatal morbidity.

1. **Objective**

The objective of this study is to evaluate the effectiveness of the guideline-implementation strategy, a decision aid (DA) for mode of delivery after previous CS, in increasing guideline adherence while not leading to a decrease in vaginal birth after caesarean (VBAC)-rate. Further, we want to evaluate the process of implementation of the DA for patients and caregivers. In addition the costs of developing and implementing the strategy will be analyzed, including the costs of a possible shift in CS rate.

1. **Trial design**

The trial design is a controlled before-and-after (CBA) trial. The intervention will be introduced on hospital level. Participating hospitals will be matched into pairs: one intervention and one control hospital.

1. **Participants and Setting**

*Hospital selection*

Twelve hospitals will be enrolled in this study. These hospitals are selected form the pool of hospitals that was enrolled previously in the SIMPLE study concerning evaluation of current care. All hospitals were matched with regard to two criteria: intended VBAC rate (≤ 60% intended VBAC (low), 60-80% intended VBAC (mean) and ≥ 80% intended VBAC (high)) and hospital type (university based hospital or community based hospital). Six matched pairs (one intervention and one control) will be selected for participation in the current study.

*Participant selection*Participants will be selected based on the following criteria:

**Inclusion criteria**

- Pregnant women who have had one previous CS
- Singleton in cephalic position
- Ability to read the Dutch language

**Exclusion criteria**

- Contra-indication for a vaginal delivery:
  - A previous classical vertical incision or other significant uterine scar
  - A previous uterine rupture
  - Suspected abruption of the placenta
  - Impossibility of vaginal birth: cervical myoma, placenta previa, vasa previa
  - Primary infection with genital herpes simplex virus (HSV)

1. **Patient recruitment and informed consent**

Since the study will be carried out on hospital-level and concerns care in accordance with evidence based guidelines, all patients who meet the study criteria will ideally receive the DA. Additionally, patients will be asked to consent for filling in questionnaires for the effect-, process- and cost-evaluation. For the questionnaires, additional inclusion-criteria apply: 1) ability to read the Dutch language and 2) aged 18 years or older. They are informed by their caregiver. The patient will be given at least one week to consider participation. After one week, when a patient is willing to participate, informed consent will be asked for and the patient will receive the digital questionnaires and in the intervention hospitals, the DA.

1. **Intervention**

The intervention consists of two elements: 1) training of healthcare professionals, 2) the DA

1) Training of healthcare professionals

All gynecologists who are employed in a hospital in which the intervention is carried out are asked to participate in this study. Researchers will train gynecologists for using the DA. Also all midwifes that relate to the involved hospitals will be asked to participate. The role of the midwifes will be to signal when to refer the pregnant woman for counseling to the hospital, to deliver the DA to the woman/couple and to shortly introduce this DA in order to prepare women/couples for the appointment with their gynecologist.

2) The patient decision Aid
The patient decision aid consists of seven steps to guide the woman/couple, together with their gynecologist, through the decision making process of mode of delivery after a previous CS. These seven steps in the DA include: 1) preference at start of counseling; 2) experience of previous delivery; 3) information about both options, about risks and benefits of both options and a prediction model for calculation the patients individual chance on a successful intended VBAC; 4) a worksheet to weigh out the options; 5) an option to discuss conditions for an intended VBAC with the gynecologist, for example: effective pain relief; 6) preliminary choice; 7) follow-up: the decision will be reevaluated. For the calculation of woman’s individual probability on a vaginal birth after previous cesarean section (VBAC) with the prediction model, a standardized computer program containing the formula will be used.

The woman/couple will be provided with the DA by their midwife or gynecologist at least two weeks before the discussion of mode of delivery is planned. Just as in usual care, the consult takes place at latest at 36 weeks gestational age (GA). During counseling, the woman/couple and gynecologist will together discuss the seven steps of the DA. In this session, a preliminary decision on mode of delivery will be made. Like in usual care, in a second session between 36 and 38 week GA, the decision on mode of delivery can be reevaluated.

1. **Control**

Usual care comprises a regular consultation with a gynecologist, without additional DA, for counseling on mode of delivery after a previous CS. This consultation usually takes place at 36 weeks GA, but local policy can vary between hospitals.

1. **Outcome measures**

Primary outcome measures

- Observed change in VBAC rate: The VBAC rate was defined as the percentage successful intended VBAC (effect measure).
- Adherence to the guideline-based quality indicators for counseling on mode of delivery after previous CS (effect measure)

Secondary outcome measures

- Decisional conflict: Decisional conflict scale (effect measure)
- Process evaluation measures:
  - Exposure to the DA and actual counseling
  - Usability, clearness of the DA
  - Women’s self-rated importance of the influence of the DA on the decision they made
  - Experience with of women and providers with Shared decision making (SDM).
  - The experiences of women and providers in terms of satisfaction with and applicability of the DA.
- Observed change in women’s preferences
- Costs analysis

1. **Data-collection**

| **Outcome measure** | **Data collection** | |
| --- | --- | --- |
| **Primary outcome measures:** | | |
| Intended VBAC rate, VBAC rate | VBAC-rate per hospital in study period, obtained retrospectively with registration of births per hospital | |
| Guideline adherence | Patient questionnaires and retrospective data-collection using case report forms (CRF’s) | |
| **Secondary outcome measures:** | | |
| Decisional conflict (DC) | A 16 item questionnaire that measures degree of uncertainty about the decision | |
| **Process evaluation:** | | |
| - Patient exposure to the DA | Retrospective data-collection with use of the registration of births per hospital and total number of inclusions | |
| - Women’s self estimated influence of the DA on decision | Questionnaire: 5-point Likert scale. After consultation but before delivery | |
| - SDM process | 9-item Shared Decision-Making Questionnaire. After consultation but before delivery | |
| - Patient satisfaction | Questionnaire  After consultation but before delivery | |
| - Provider satisfaction | Depth interviews of 20-30 minutes during intervention study period in the participating hospitals  Obtained in all participating intervention hospitals | |
| **Other secondary outcome measures:** | | |
| Preferences in mode of delivery | Questionnaire. After consultation but before delivery. | |
| Usability, clearness of the DA | Questionnaire. After consultation but before delivery | |
| **Cost-analysis** | | |
| 1) the costs of the development of implementation strategy | Development costs: time investment of members of project group and experts. Printing costs |  |
| 2) Implementation costs of the strategy | Interviews with professionals for estimation of extra consultation time in intervention group. Also, an attendance list with names and function of professionals attending the training. The duration of each training will be registered. Also the time investment of the trainer will be accounted for. |  |
| 3) the costs of a shift in CS rate | Resource use will be derived from the Case Report Form (CRF).   - Total number of consultations with the gynaecologist after 38+6 weeks gestational age (GA). - Development of severe morbidity after 39 weeks GA: intra uterine foetal demise (IUVD), HELLP/ preeclampsia/ eclampsia / other - Actual mode of birth   During labor   - Duration admission labour room - Epidural - Interventions: method of induction, intrapartum antibiotics, manual removal placenta, instrumental delivery, suture in operation room (OR), embolisation,   Maternal data:   - Hospital days   Nursing unit: ward / medium care / intensive care   - Complications (until 6 weeks postpartum): uterine rupture, hysterectomy, operation trauma, curettage, re-operation, thrombo-embolic complications, maternal death   Neonatal data:   - Hospital days   Nursing unit: ward / medium care / high care / neonatal intensive care unit (NICU)   - Complications (until 6 weeks after birth): death, pH< 7.10 or BE -12.5 or APGAR after 5 min <7 |  |

1. **Sample size**

A non-inferiority limit of 10% was chosen. This implies that a decrease of 10% or more in VBAC rate will be seen as inferior care in the intervention group in comparison to the non-intervention group. Previous study results of the SIMPLE study showed an intended VBAC rate of 67% with a success rate of 72% in 2010, therefore we assume the baseline VBAC rate to be 48.2%. In absolute numbers, a VBAC rate of 38.2% in the intervention group would be seen as ‘inferior’ care. For calculation of the sample size in order to show non-inferiority, we chose an alpha of 0.05 and a beta of 0.20, we estimated the inter correlation coefficient (ICC) to be 0.2. The estimated sample size, corrected for cluster variation, was 400 per arm.

With regard to primary outcome measure one, VBAC-rate, the total estimated sample size is 800 women, this sample size was also found to be sufficient for obtaining the secondary outcome measures. Previous study results showed that of 11,7% of the pregnant women in the Dutch hospitals have a history of CS and approximately in total 7% are suitable for inclusion. With a mean of 1000 labours per hospital per year, we can include 70 women per hospital per year, therefore the approximated duration of the study is 12 months.

1. **Data-management**

Informed consent forms are filled in before the start of the online-questionnaire. Questionnaires will be filled and saved via an online questionnaire system (surveymonkey). CRF’s will be stored in an excel-database. In order to maintain privacy of the participants, all patient data will be filed in the database using subsequent case numbers. A patient identification list will be maintained in a separate, locked, excel-file.

1. **Data analysis**

Data analysis will be carried out with SPSS 19.0 and Atlas.

- Intended VBAC rate, repeat CS rate, VBAC rate will be obtained by calculating the mean difference in percentage between the intervention group and the non-intervention group.
- Guideline adherence: the observed change in guideline adherence between the time-period before and after implementation of the DA. This will be calculated per hospital and in total. Percentages guideline adherence before and after the implementation strategy will be compared by using crosstabs.
- Decisional Conflict Score will be compared between the intervention group and the non-intervention group. . To calculate the DC score, first the 16 items of the DC score are summed. The items are scored as follows: 0=strongly agree; 1=agree; 2=neither agree nor disagree; 3=disagree and 4=strongly disagree. The sum is divided by 16 and multiplied by 25. Scores range from 0 (no decisional conflict) to 100 (extremely high decisional conflict) [4]. Total scores will be compared.
- Process evaluation:
  - Introduction of the DA and training of the Providers Aspects of environment that might influence implementation of the DA[12] will be examined in focus groups. Data will be analyzed by using Atlas.
  - Exposure to the DA and actual counseling: percentage of women that did receive the DA and whether the DA was discussed with the gynecologist during the intervention.
  - The experiences of women in terms of satisfaction, applicability and clarity will be examined by using a questionnaire.
  - The experiences of providers in terms of satisfaction, applicability and clarity will be examined in a qualitative study by using focus groups during and at the end of the inclusion period Data will be analyzed by using Atlas. Women’s self-rated importance of the influence of the DA on the decision they made: 5 point Likert scale. (1=not important for the decision, 5= very important for the decision).
  - SDM process and the influence of the DA on this process: 9-item Shared Decision-Making Questionnaire during the intervention.
- Costs analysis: the cost-analysis consists of three aspects: 1) the costs of the development of the implementation strategy; 2) the costs of implementing the strategy and 3) the costs of shift in CS rate

1. **Ethical considerations**

Informed consent will be asked for filling in one questionnaire and for whether the woman approves to be contacted by the researchers for additional information. Filling in the questionnaires is expected to have neither a great impact on these women nor to be very time-consuming.

Care with use of the DA is considered regular care. However, women can opt not to use the DA when their hospital is enrolled in the study as they have the right not to be informed. This option is communicated to all participating gynaecologist during the training session and it is described in a user manual for the DA.

The Medical Ethical Committee (CMO) of Maastricht university medical centre (MUMC) declared that this study can be seen as ‘regular care’ since all elements involved are included in the guideline of the Dutch organization of gynecology and obstetrics (NVOG): ‘pregnancy and labour after previous caesarean section 04-06-2012’.

**References**

[1] Stacey D, Bennett CL, Barry MJ, Col NF, Eden KB, Holmes-Rovner M, Llewellyn-Thomas H, Lyddiatt A, Légaré F, Thomson Ra: Decision aids for people facing health treatment or screening decisions. Cochrane Database of Systematic Reviews 2011, Issue 10.

[2] Shorten A, Shorten B, Keogh J, West S, Morris J. Making choices for childbirth: a randomized controlled trial of a decision-aid for informed birth after cesarean. *Birth* 2005; **32**(4):252–61.

[3] Montgomery AA, Emmett CL, Fahey T, et al. Two decision aids for mode of delivery among women with previous caesarean section: randomized controlled trial. BMJ 2007;334:1305.

[4] Ottawa Health Decision Centre. User manual—decisional conflict scale(s)

[5] National Institutes of Health Consensus Development conference statement: vaginal birth after cesarean: new insights March 8-10, 2010. Obstet Gynecol. 2010 Jun;115(6):1279-95

[6] Kwee A, Bots ML, Visser GH, Bruinse HW. Obstetric management and outcome of pregnancy in women with a history of caesarean section in the Netherlands. Eur J Obstet Gynecol Reprod Biol. 2007 Jun;132(2):171-6.

[7] McMahon MJ, Luther ER, Bowes WA, Jr., Olshan AF. Comparison of a trial of labor with an elective second cesarean section. N Engl J Med. 1996 Sep 5;335(10):689-95.

[8] Grobman WA, Lai Y, Landon MB, Spong CY, Leveno KJ, Rouse DJ, et al. Does information available at admission for delivery improve prediction of vaginal birth after cesarean? Am J Perinatol. 2009 Nov;26(10):693-701.

[9] Grobman WA, Lai Y, Landon MB, Spong CY, Leveno KJ, Rouse DJ, et al. Development of a nomogram for prediction of vaginal birth after cesarean delivery. Obstet Gynecol. 2007 Apr;109(4):806-12.

[10] Costantine MM, Fox KA, Pacheco LD, Mateus J, Hankins GD, Grobman WA, et al. Does information available at delivery improve the accuracy of predicting vaginal birth after cesarean? Validation of the published models in an independent patient cohort. Am J Perinatol. 2011 Apr;28(4):293-8.

[11] Costantine MM, Fox K, Byers BD, Mateus J, Ghulmiyyah LM, Blackwell S, et al. Validation of the prediction model for success of vaginal birth after cesarean delivery. Obstet Gynecol. 2009 Nov;114(5):1029-33

[12] Saunders RP, Evans, MH, Joshi P, Developing a Process-Evaluation Plan

for Assessing Health Promotion Program Implementation: A How-To Guide, [Health Promot Pract.](http://www.ncbi.nlm.nih.gov.ezproxy.ub.unimaas.nl/pubmed?term=Saunders RP%2C Evans%2C MH%2C Joshi P%2C Developing a Process-Evaluation Plan) 2005 Apr;6(2):134-47
