# Supplementary material for: The effect of the use of a decision aid with individual risk estimation on the mode of delivery after a caesarean section: A prospective cohort study
Source: PLoS One. 2019 Sep 26;14(9):e0222499. doi: 10.1371/journal.pone.0222499 (PMC6763212; doi:10.1371/journal.pone.0222499)
Supplement: S1 Table — (DOCX) [file pone.0222499.s004.docx]

S1. Table Overview of VBAC-rates before and after the intervention per hospital

| **Hospital** | **VBAC rate before** | **VBAC rate after** |
| --- | --- | --- |
| **Intervention group** |  |  |
| NUT hospital | 75% | 51,9% |
| NUT hospital | 45,87% | 43,6% |
| NUNT hospital | 43,63% | 53,9% |
| NUNT hospital | 30,8% | 38,1% |
| U hospital | 36,64% | 49,0% |
| NUT hospital | 57,1% | 32,0% |
| **Control group** |  |  |
| U hospital | 54,3% | 48,2% |
| NUT hospital | 72,2% | 41,6% |
| NUNT hospital | 58,8% | 50% |
| NUNT hospital | 27,90% | 31,2% |
| NUT hospital | 55,56% | 43,8% |
| NUT hospital | 63,4% | 53,8% |

NUT: non-university teaching, NUNT: non-university non-teaching, U: university
